# A cross-sectional survey on health care professionals’ approaches, challenges, and support needs when addressing life threat with recipients of an allogeneic hematopoietic stem cell transplantation and with their relatives

**DOI:** 10.1007/s00277-025-06211-6

**Published:** 2025-02-08

**Authors:** Anne Pralong, Steffen T. Simon, Udo Holtick, Alinda Reimer, Berenike Schoerger, Sukhvir Kaur, Jithmi Weliwitage, Martin Hellmich, Michael Hallek, Christof Scheid, Raymond Voltz, Marco Herling

**Affiliations:** 1 University of Cologne, Faculty of Medicine and University Hospital, Department of Palliative Medicine, Cologne, Germany; 2 University of Cologne, Faculty of Medicine and University Hospital, Centre for Integrated Oncology Aachen-Bonn-Cologne-Duesseldorf (CIO ABCD), Cologne, Germany; 3 University of Cologne, Faculty of Medicine and University Hospital, Centre for Health Services Research (ZVFK), Cologne, Germany; 4 University of Cologne, Faculty of Medicine and University Hospital, Department I of Internal Medicine, Cologne, Germany; 5 University of Cologne, Faculty of Medicine and University Hospital Cologne, Institute of Medical Statistics and Computational Biology (IMSB), Cologne, Germany; 6 Department of Hematology, Cellular Therapy, Hemostaseology, Infectious Diseases, University of Leipzig, Leipzig, Germany; 7 Cancer Center Central Germany (CCCG) Leipzig-Jena, Leipzig, Germany

**Keywords:** Stem cell transplantation, Allogeneic transplantation, Health care professionals, Attitudes towards death

## Abstract

Patients treated with an allogeneic hematopoietic stem cell transplantation (allo-SCT) face a significant mortality risk, which might trigger existential distress. We aimed to investigate how hematology health care professionals (HCPs) address life-threatening conditions with allo-SCT recipients and their relatives, and what are challenging and supportive aspects. We conducted a multicenter cross-sectional survey, assessing: (1) HCPs’ approaches to address life threat, using a self-developed questionnaire with 19 items categorized in four dimensions (proactive, cautious, reactive, avoiding), (2) challenges/support needs, using self-developed multiple-choice questions, and (3) personal attitudes towards death (Death Acceptance subscale of the Life Attitude Profile-Revised; Death Attitude Profile-Revised). Statistics included association, regression and exploratory factor analysis. We included 104 participants (52 physicians, 45 nurses, 7 psycho-oncologists) from the settings of SCT, general hematology and intensive care. Physicians predominantly addressed life threat proactively, while nurses tended to raise the topic when prompted by patients or a clinical worsening. Overall, 10% of HCPs stated to avoid the topic, even in case of a high risk of death. HCPs’ approaches correlated weakly with their attitudes towards death. A main challenge was to talk with patients/relatives repressing the risk of death (72%). More time resources for these conversations (71%), and mentoring by palliative-care specialists (67%) or psycho-oncologists (65%) were the most frequent support needs. This comprehensive survey reveals strong differences between professions in addressing life threat, and possible influence from personal death attitudes. Further confirmatory research in larger samples is needed to tailor communication trainings on life threat in multi-professional teams. Registration:German Clinical Trials Register, DRKS00027290, date of registration: 2022-01-10.

**Supplementary Information:**

The online version contains supplementary material available at 10.1007/s00277-025-06211-6.

## Introduction

The five-year survival after an allogeneic hematopoietic stem cell transplantation (allo-SCT), all indications included, is about 50% and attributed to progression or relapse of the hematologic disease and the toxicity of the procedure [1–3]. Allo-SCT recipients face, therefore, a ‘coexisting’ chance of cure and a relevant risk of dying. Facing existential threats may lead to existential concerns [4, 5]: some studies have highlighted clinically relevant existential distress related to fear of recurrence [6], fear of death [7, 8], or uncertainty of the future [9]. However, little is known on how health care professionals (HCPs) raise these existential issues, especially in the allo-SCT setting.

In general cancer care, various attitudes adopted by HCPs to address life-threatening issues have been described, ranging from self-confidence [10] and directness in the style of communication [11], up to attitudes of avoidance [12, 13], and even withholding information from patients [11, 12]. Barriers for raising existential issues were attributed to feelings of powerlessness, identifying oneself with patients [14], fear of diminishing hope, or time constraints [15]. Personal attitudes toward death may also represent a barrier [11, 16, 17].

In the hematology setting, hematologists appear to be more reluctant than oncologists treating patients with solid tumors, to engage in discussions on prognosis [18], they employ more general terms, or they address the topic only once [19]. As described in the literature, a reason for this could be, in addition to barriers mentioned above, the prognostic uncertainty, which often characterizes the course of hematological malignancies [18, 20]. Not only physicians, but also patients may show more ambivalence: different wishes for prognostic information have been highlighted [18]. Gray et al. hypothesize that these differences might be precisely due to the prognostic uncertainty, but also to the heterogeneous nature of hematological diseases [18].

Therefore, the threat to life associated with an allo-SCT, where the chance for cure and the risk of death coexist closely, appears to give rise to varying burdens and needs among patients, and possibly their relatives: existential distress, different or ambivalent demand for prognosis information. To adequately meet these burdens and needs in the clinical setting, it is crucial to better understand how HCPs themselves approach life-threatening issues. With this study, we aimed to investigate how HCPs address life threat with allo-SCT recipients and their relatives, which approaches they adopt and what their challenges and support needs are.

## Materials and methods

This quantitative cross-sectional survey is part of the research project “Allo-PaS” (Palliative-supportive management in allo-SCT; registration number: DRKS00027290, German Clinical Trials Register) [4]. Findings are reported following the STROBE Statement for cross-sectional studies [21].

### Design

The online survey was sent to five German tertiary hospitals, all part of comprehensive cancer centers (CCC): The University Hospitals of Aachen, Bonn, Cologne, and Düsseldorf, joined in the Centre for Integrated Oncology (CIO ABCD), and the University Hospital of Leipzig. A local person from each SCT department was contacted, who consequently forwarded the survey to HCPs based in his/her center. They had to be older than 18 years, to be a physician, a nurse or a psycho-oncologist; to be involved in the care of patients before, during or after an allo-SCT, in an out- or inpatient setting; to have given their informed consent; and to be proficient in German. Data were collected from January to February 2023 using LimeSurvey^®^. Provision of psycho-oncology care and specialist palliative care for allo-SCT recipients was guaranteed in all five centers.

### Establishment

The self-developed questions based on preliminary qualitative results of the Allo-PaS research program [4], on a literature review [5] and on iterative discussions between a researcher (AP), a palliative-care physician (StS) and two hematologists/transplanters (UH, M. Herling). It was piloted for comprehensibility, readability, plausibility and work effort among 10 researchers and HCPs from different professions and disciplines: allo-SCT, general oncology and hematology, psycho-oncology and palliative care; and then refined based on respective feedback.

The survey consisted of three sections, additionally to demographics: (1) how HCPs address life threat with patients in a situation of a concomitant chance of cure and a relevant risk of death, (2) what are challenges and support needs, and (3) personal attitudes towards death. Details are listed in Table 1 and Online Resource 1.

**Table 1:** Survey structure

Development type	Question type	Number of items	Dimensions	
**1. HCPs’ approaches when addressing the threat to life**				
Self-developed	5-point Likert scale (1, “I don’t agree at all” to 5, “I strongly agree”)	19	Proactive approach	Addressing proactively the life threat by giving patients/relatives the opportunity to talk about it. (This approach assumes respect for the patient’s wishes, particularly their wish not to talk about this topic).
			Reactive approach	Addressing the life threat when prompted by the patients themselves or by a worsening of the clinical situation
			Cautious approach	Being deliberately reluctant to address the life threat in moments of greater vulnerability, with the aim to preserve hope in the patients
			Avoiding approach	Avoiding the topic altogether
			Time constraint (single item)	
**2. HCPs’ challenges and needs for support when discussing the threat to life**				
**Challenges**				
Self-developed	Multiple choice	10 (+ 1 free-text answer for further challenges)		
**Needs for support**				
Self-developed	Multiple choice	10 (+ 1 free-text answer for further needs)		
**3. Personal attitudes toward death**				
***Death Attitude Profile-Revised*(DAP-R)** [22, 23]				
Standardized, validated	7-point Likert scale (1, “strongly disagree” to 7, “strongly agree”)	32	Fear of death	Negative thoughts and feelings about death and dying
			Death avoidance	Avoiding thoughts about death to reduce death anxiety
			Neutral acceptance	View of death as something that is neither feared nor welcomed
			Approach acceptance	View of death as a gateway to a positive afterlife
			Escape acceptance	View of death as an escape from a painful existence
‘**Death Acceptance**’ **subscale of the*Life Attitude Profile-Revised*(LAP-R)** [24, 25]				
Standardized, validated	7-point Likert scale (1, “strongly disagree” to 7, “strongly agree”)	8	-	
**4. Demographics and clinical characteristics**				
Age, mother tongue, gender, profession, years of clinical practice with allo-SCT patients, annual number of allo-SCT patients and annual number of patients cared for during their dying phase				

## Results

The characteristics of the 104 included HCPs are presented in Table 2. The invitation link was opened by 184 HCPs. Out of them, 76 were considered unit non-response, as they did not fill in the questions included in this analysis, and four did not fulfil the inclusion criteria: These were all excluded from the analysis. One participant dropped out before answering the LAP-R questionnaire. The optional DAP-R questionnaire was completed by 81 participants. Due to confidentiality preferences of some centers, the number of HCPs, to whom the survey was forwarded, was not disclosed, so the exact response rate is not available. Results of the exploratory analysis are presented in Online Resource 2.

**Table 2:** HCPs’ demographics and clinical characteristics

Characteristics (*N* = 104)					No. (%)
Gender (female)					61 (58.7)
Age (years; mean and standard deviation)					37.2 (8.1)
Mother tongue					
German					100 (96.2)
Other					4 (3.8)
Profession and health care service					
	*SCT* ^1^	*General hematology* ^1,2^	*ICU*	*Not stated*	*Total*
Nurse	30	13	0	2	45 (43.3)
Physician	24	19	9	-	52 (50.0)
Psycho-oncologist^*^	5	2	-	-	7 (6.7)
Years of clinical practice with allo-SCT patients (median and range)					6.5 (0–25)
No. of allo-SCT patients per year (median and range)					47.5 (1-350)
No. of patients cared for in the dying phase per year (median and range)					5.0 (0-100)

^1^ In- and/or outpatients

^2^ Physicians and nurses had training in general oncology, hematology and transplantation.

### Statistics

Descriptive statistics included frequencies, means, and medians. Associations between nominal variables were calculated using Chi^2^ or Fisher’s exact tests, with further post-hoc tests to determine column proportions (Bonferroni tests). For metric variables, Spearman’s rho correlations were ascertained. Mann-Whitney-U/Kruskal-Wallis tests for independent samples were carried out for associations between metric and nominal variables. We entered selected demographics in multiple linear regression analyses to find insights in their effect on the dependent variables.

For HCPs’ approaches (survey part 1), we calculated the frequency of consented statements, defined as the choice for ‘I strongly agree’ or ‘I rather agree’. Furthermore, we conducted an exploratory factor analysis to test whether the four approaches built a priori on a theoretical basis could be confirmed statistically (Online Resource 2). After confirmation, four subscales, based on the final factor analysis, were determined by calculating their means.

Analyses were considered statistically significant if the two-sided *p*-value was < 0.05. We used SPSS (Version 28.0.1.0) for all analyses.

### HCPs’ approaches when addressing life threat

Table 3 lists the single items of the questionnaire with frequency of agreement.

**Table 3:** Questionnaire on how HCPs approach the topic of life threat

	Professional groups			
	Total **(*N*** **= 104)**	Physicians **(*n*** **= 52)**	Nurses **(*n*** **= 45)**	Psycho-oncologists **(*n*** **= 7)**
**Items**	Count (% of total)^#^	Count (% within profession)^#^		
*Proactive approach*				
It is important to me to involve my patients’ relatives in the conversation about the risk of dying.	71 (68%)	43 (83%)_a_	25 (56%)_b_^**^	3 (43%)_a, b_
It is important to me to inform all patients well about their risk of dying.	69 (66%)	46 (89%)_a_	21 (47%)_b_^***^	2 (29%)_b_^***^
If there is a high risk of death (80–90%, e.g. second allo-SCT), I try to talk to the patient/relatives to help them prepare for the possibility of dying.	59 (57%)	34 (65%)	19 (42%)	6 (86%)
I repeatedly raise the topic with patients/relatives who unrealistically overestimate the prognosis.	51 (49%)	37 (71%)_a_	13 (29%)_b_^***^	1 (14%)_b_^**^
To give a realistic picture and prevent false hopes, I deliberately talk about this topic in advance of a planned SCT.	50 (48%)	39 (75%)_a_^***^	7 (16%)_b_	4 (57%)_a_^*^
I always discuss worst and best-case scenarios with the patients.	47 (45%)	38 (73%)_a_	9 (20%)_b_^***^	0^1^
I broach the topic with all patients/relatives, unless they expressly do not wish it.	43 (41%)	29 (56%)_a_	12 (27%)_b_^*^	2 (29%)_a, b_
If there is a high risk of death (80–90%, e.g. second allo-SCT), I do not want to weaken the patients’/relatives’ hope of cure and therefore deliberately do not talk about this topic. (inverse item)	10 (10%)	1 (2%)_a_	8 (18%)_b_^*^	1 (14%)_a, b_
*Cautious approach*				
During the stressful time of allo-SCT, I avoid this topic in order to give patients confidence.	38 (37%)	20 (39%)	17 (38%)	1 (14%)
I try to emphasize the chance of cure when talking to particularly worried patients/relatives.	31 (30%)	17 (33%)	14 (31%)	0^1^
*Reactive approach*				
I only discuss this topic once the patients/relatives bring it up themselves.	29 (28%)	3 (6%)_b_	22 (49%)_a_^***^	4 (57%)_a_^***^
I only address this topic once there is a high risk of death (80–90%, e.g. second allo-SCT).	20 (19%)	6 (12%)	13 (29%)	1 (14%)
I only address this topic once a planned change of goal of care (curative to palliative) is pending.	17 (16%)	0^1^	17 (38%)	0^1^
*Avoiding approach*				
I struggle to talk to patients/relatives about this topic.	11 (11%)	3 (6%)	8 (18%)	0^1^
I avoid this topic because it causes me anxiety and/or insecurity.	8 (8%)	2 (4%)	6 (13%)	0^1^
After I or others have communicated the risk of death to the patients/relatives, I avoid asking what triggers it in them.	5 (5%)	4 (8%)	1 (2%)	0^1^
I prefer to avoid this topic with patients/relatives, even if I know that they need to talk about it.	4 (4%)	1 (2%)	3 (7%)	0^1^
It is not my role to talk about this topic.	3 (3%)	0^1^	3 (7%)	0^1^
*Other (time constraint)*				
I don’t have the time to discuss this topic in depth, so I often don’t.	21 (20%)	7 (14%)_b_	14 (31%)_a_^*^	0^1^

^#^Frequency (count and percent) of consented items (= I rather agree + I strongly agree) in total and within each professional group

a, b, c = denote a significant group difference if the letters a, b or c differ between groups with: * *p* ≤ 0.05; ** *p* ≤ 0.01; *** *p* ≤ 0.001. Difference is measured by Bonferroni tests

^1^ This category is not used in comparisons because its column proportion is equal to zero or one

#### Proactive approach

At the subscale level, HCPs embraced mainly a proactive approach (*Mdn* = 3.4, range 0.7-5.0, Table 4). However, considering specific items, only a slight majority (57%) agreed rather/strongly that they would ‘try to talk to the patients/relatives to help them prepare for the possibility of dying’ in case of a high mortality risk of 80–90%. 10% stated they do ‘not want to weaken the patients’/relatives’ hope of cure and therefore deliberately do not talk about this topic’.

**Table 4:** Median values of HCPs’ approaches to life threat and associations with profession/gender

Approach^1^	Profession						Gender		
	*Median (range)*				*Kruskal-Wallis test*		*Median (range)*		*Mann-Whitney-U test*
	Total (*N* = 104)	Nurse (*n* = 45)	Physician (*n* = 52)	Psycho-oncologist (*n* = 7)	*H-*value (*df*)	Pairwise comparison	Female (*n* = 61)	Male (*n* = 43)	*z-*value
Proactive approach	3.4 (0.7-5.0)	2.9 (0.7–4.4)	4.0 (2.4-5.0)	2.6 (1.6–4.1)	26.8 (2) ^***^	Phy > Nur, Psy	3.1 (0.7–4.9)	4.0 (1.4-5.0)	4.4^***^
Cautious approach	3.0 (0.5-5.0)	3.0 (0.5-5.0)	3.0 (1.0-4.5)	2.5 (2.0-3.5)	0.9 (2)		3.0 (1.0–5.0)	3.0 (0.5–4.5)	1.4
Reactive approach	2.3 (1.0-4.7)	3.0 (1.3–4.7)	1.7 (1.0-3.7)	2.7 (1.3-4.0)	35.7 (2) ^***^	Nur > Phy	2.7 (1.0-4.7)	2.0 (1.0-3.7)	3.8 ^***^
Avoiding approach	1.8 (1.0-3.8)	2.0 (1.0-3.8)	1.5 (1.0-3.7)	1.5 (1.0–2.0)	19.7 (2) ^***^	Nur > Phy	1.8 (1.0-3.8)	1.5 (1.0-3.5)	2.6 ^**^

^1^ Based on the subscales built by exploratory factor analysis (Online resource 2)

* *p* ≤ 0.05; ** *p* ≤ 0.01; *** *p* ≤ 0.001

Abbreviations: Nur = nurse; Phy = physician; Psy = psycho-oncologist

##### Differences among HCP subsets and associations

We identified substantial differences between professional groups. At the subscale level, physicians adopted a more proactive attitude than nurses (*p* < 0.001) and psycho-oncologists (*p* = 0.002; Table 4). The proactive approach was also the one physicians assumed most often, compared to other approaches (*Mdn* = 4.0, range 2.4-5.0, Table 4). At the level of single items, physicians often agreed they ‘always discuss best and worst-case scenario’ (73%), discuss the risk of dying in advance of an allo-SCT (75%) and ‘repeatedly raise the topic with patients/relatives who unrealistically overestimate the prognosis’ (71%). The differences to nurses were significant (*p* < 0.001). Furthermore, 18% of nurses rather/strongly agreed they ‘deliberately do not talk about this topic’ if there is a high risk of death. This was significantly more than for physicians (*p* = 0.02; Table 3). However, as the small sample size limits statistical power and harbors risks for bias, these findings need confirmation in larger cohorts.

HCPs caring annually for more dying patients were also more proactive (*r* = 0.229, *p* = 0.019; Table 5). In the subgroup analysis, this correlation was confirmed for physicians only (Online Resource 3). Regarding personal death attitudes, two DAP-R subscales, approach acceptance (*r* = -0.224, *p* = 0.044) and escape acceptance (*r* = -0.244, *p* = 0.028), negatively correlated with the proactive approach in the total sample (Table 5), but not in the subgroup analysis. There was no significant association with gender (Table 4) in the regression model (Online Resource 4).

**Table 5:** Correlations between HCPs’ approaches to life threat and demographics/personal attitudes towards death

	Proactive approach	Cautious approach	Reactive approach	Avoiding approach
**Demographics** (*N* = 104)				
Age	n.s.	n.s.	n.s.	n.s.
Years of clinical practice with allo-SCT patients	n.s.	0.208^*^	n.s.	n.s.
No. allo-SCT patients per year	n.s.	0.196^*^	n.s.	n.s.
No. patients cared for in the dying phase per year	0.229^*^	n.s.	n.s.	n.s.
- dying phase without outliers (*n* = 95)	0.256^*^	n.s.	-0.208^*^	-0.215^*^
**Personal attitudes towards death**				
LAP-R (*n* = 103)	n.s.	n.s.	n.s.	-0.206^*^
DAP-R (*n* = 81):				
Fear of death	n.s.	n.s.	n.s.	n.s.
Death avoidance	n.s.	0.221^*^	0.275^*^	n.s.
Neutral acceptance	n.s.	n.s.	n.s.	n.s.
Approach acceptance	-0.224^*^	n.s.	0.243^*^	n.s.
Escape acceptance	-0.244^*^	n.s.	n.s.	n.s.
**Intercorrelation** (*N* = 104)				
Cautious approach	n.s.			
Reactive approach	-0.582^***^	0.318^***^		
Avoiding approach	-0.416^***^	n.s.	0.518^**^	

Approach subscales built by exploratory factor analysis (Online resource 2)

* *p* ≤ 0.05; ** *p* ≤ 0.01; *** *p* ≤ 0.001; n.s. = not significant

#### Cautious approach

The corresponding subscale was the second most frequent approach adopted in the total sample (*Mdn* = 3.0, range 0.5-5.0, Table 4). At the item level, when facing particularly vulnerable patients, about one third of HCPs agreed that they remain reluctant about addressing life threat and rather try to preserve hope (Table 3).

##### Differences among HCP subsets and associations

There were no significant differences between professional groups in terms of the frequency with which they embraced a cautious approach (Table 4). A weak correlation with years of practice with allo-SCT patients (*r* = 0.208, *p* = 0.035) and number of patients per year (*r* = 0.196, *p* = 0.048) indicated that more experienced HCPs adopted also a more cautious approach. We further found a weak positive correlation with the DAP-R subscale of death avoidance in the total sample (*r* = 0.221, *p* = 0.049; Table 5), but not among HCP subsets (Online Resource 3).

#### Reactive approach

This approach was the third most frequent one in the total sample (*Mdn* = 2.3, range 1.0-4.7, Table 4). At the item level, about one third of HCPs would ‘only discuss life threat once the patients or relatives bring it up themselves’. A smaller proportion of HCPs would only address the topic once the risk of dying is very elevated (19%) or a change in goals of care is pending (16%) (Table 3).

##### Differences among HCP subsets and associations

Nurses adopted this approach more frequently than physicians at the subscale level (*p* < 0.001, Table 4). It was also the one nurses most used, together with the cautious approach, as compared to other approaches (*Mdn* = 3.0, range 1.3–4.7, Table 4). At item level, nurses, but also psycho-oncologists, were more inclined than physicians to wait until patients or relatives bring the topic up themselves (*p* = 0.000). Although the difference was not significant, 12% of physicians and 29% of nurses would wait until the risk of death is very elevated before addressing it. Only nurses would wait until a change in goals of care is pending (Table 3).

We found a weak negative correlation with the annual number of patients cared for in the dying phase after adjusting for outliers (*r* = -0.208; *p* = 0.043). As for the cautious approach, a higher DAP-R attitude of death avoidance (*r* = 0.275; *p* = 0.013) and approach acceptance (*r* = 0.243; *p* = 0.029) weakly correlated with a more reactive approach (Table 5). The subgroup analysis confirmed this finding only for death avoidance in physicians (Online Resource 3). The significant association with gender was not found in the regression model (Online Resource 4).

#### Avoiding approach

The questionnaire items with the lowest consent reflected an avoiding approach. Overall, 8% of HCPs acknowledged to avoid the topic because of anxiety or feeling insecure; 4% stated to avoid the topic even if they would know about the patients’ or their relatives’ wish to talk about it.

##### Differences among HCP subsets and associations

At the subscale level, the avoiding approach was more common among nurses than physicians (*p* < 0.001; Table 2). At the item level, no psycho-oncologist agreed upon any of the items (Table 3). Furthermore, HCPs with less experience with dying patients embraced the avoiding approach, although the correlation was weak and after adjusting for outliers (*r* = -0.215, *p* = 0.037). There was a weak negative correlation with death acceptance (LAP-R; *r* = -0.206, *p* = 0.036; Table 5), which was no longer observed at the level of professional subsets (**Online Resource 3**).

#### Time constraint

More nurses (31%) than physicians (14%, *p* = 0.035) rather/strongly agreed that they ‘don’t have the time to discuss this topic in depth, so [they] often don’t’ (Table 3).

### HCPs’ challenges and support needs to address life threat

#### Challenges

**Fig. 1:**
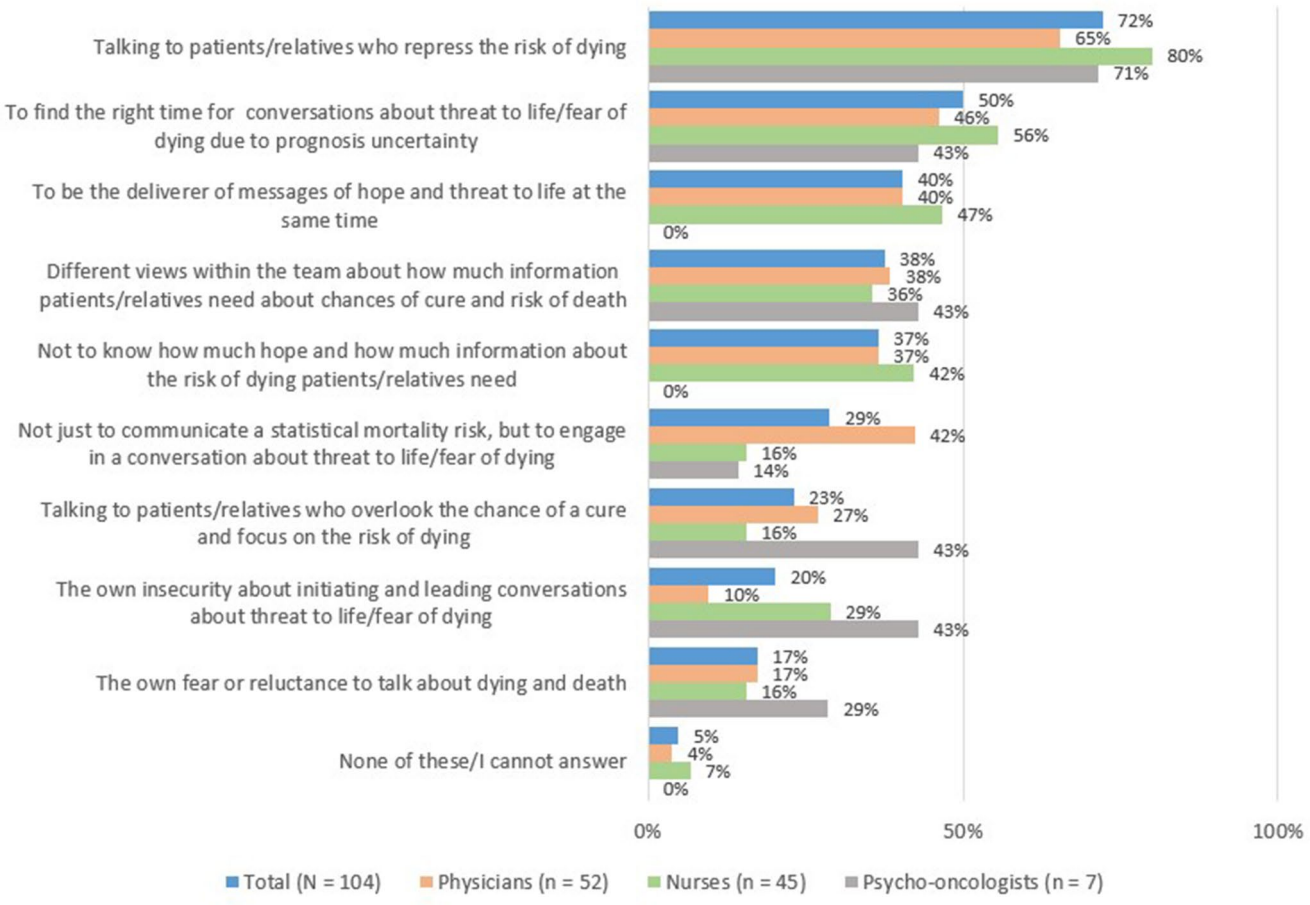
Challenges when addressing life threat (frequency of participants in total and within each professional group in %)

The primary challenge stated by 72% of HCPs was communicating with patients/relatives who repress the risk of dying, with no significant differences between professions. Further common challenges were to find the right timing for discussions about life threat, due to prognosis uncertainty (50%), and to be the deliverer of messages of hope and threat at the same time (40%). Internal inhibitions such as the own fear and the insecurity to talk about death were the least challenging issues (Fig. 1).

Insecurity in talking about life threat was stated less often as a challenge by physicians than by nurses or psycho-oncologists (*p* = 0.019; Online Resource 5) and was associated with less experience with dying patients (*p* = 0.020; Online Resource 6). ‘Finding the right timing for such conversations due to prognosis uncertainty’ was more challenging for younger participants (*p* = 0.003; Online Resource 6).

#### Needs for support

**Fig. 2:**
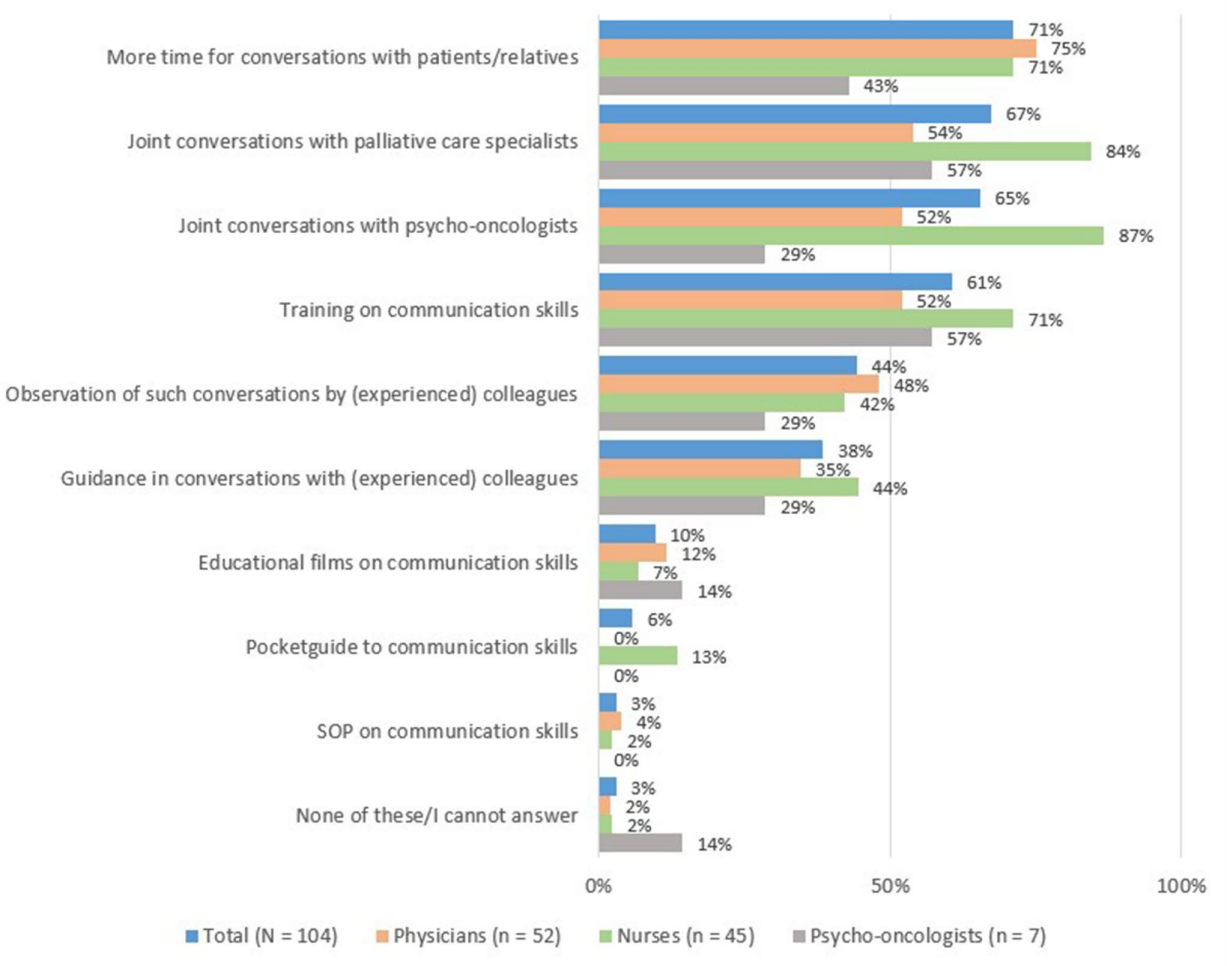
Supportive offers that HCPs wish for conversations on life threat (frequency of participants in total and within each professional group in %)

More time for conversation was the most common identified need of support (71%; Fig. 2). Personalized supportive offers were strongly preferred as compared to general information material like films, Standard Operating Procedures (SOP), or pocket guides. Among personalized offers, participants, especially nurses (Online Resource 7), favored help from outside the transplantation team, like conversations together with palliative-care specialists or psycho-oncologists. Mentoring by experienced team members, like observation of or guidance on conversations on life threat, were less often prioritized, and associated with a younger age and less clinical experience (Online Resource 8).

There were only few free-text answers to the question about further challenges, respectively support needs, and these did not raise any new relevant topics.

## Discussion

To our knowledge, this is the first report of a cross-sectional survey on HCPs’ preferences in dealing with patient’s existential threats in an allo-SCT setting. Here, most HCPs adopted a proactive approach when addressing the topic. However, there were substantial differences between professional groups. Furthermore, a non-negligible proportion of HCPs stated to avoid discussing the topic, even in case of a high risk of death. The main challenge for HCPs was to talk with patients or relatives who repress the risk of dying. More available time and mentoring by palliative-care specialists or psycho-oncologists in discussing life-threatening situations emerged as central support needs.

Physicians adopted predominantly a proactive approach. We interpret this primarily as an expression of their genuine responsibility to disclose prognosis and to inform on the risk of dying. In contrast, a fundamental avoiding approach was rare. Yet, about one fourth of participating physicians stated only unfrequently to systematically discuss best and worst-case scenarios or to re-address the topic of life threat with patients/relatives who unrealistically overestimate the prognosis. Discussing best and worst-case scenarios has been described as an important tool to deal with prognostic uncertainty and help patients to understand their prognosis, to give informed consent and to be better prepared for the future [18]. Re-addressing the risk of dying in the course of the disease also plays an important role when discussing prognosis [19]. Both communication tools– discussing worst and best-case scenario and revisiting the risk of dying– are useful in meeting the specific challenges of the allo-SCT setting, where the prognostic uncertainty is particularly pronounced. They can be easily implemented in clinical practice and communication trainings.

In our cohort, nurses were overall less proactive. We hypothesize that this is primarily due to their professional role that does not imply disclosing prognosis. Yet, only 7% of nurses rather/strongly agreed that talking about life threat was not part of their role. This consciousness that addressing existential issues in general, beyond mere prognostic disclosure, is part of nurses’ area of responsibility corroborates with other results [15].

The reactive approach was frequent among nurses. For instance, half of them stated to wait until the patients/relatives bring the topic up themselves. This might reflect the fact that patients can tend to talk to nurses first before approaching physicians, as suggested elsewhere [26, 27]. Another hypothesis could also be that the unique proximity to patients leads more easily to emotionally laden situations, which may result in turn in avoiding attitudes [28]. This can also partly explain that participating nurses tended to a more avoiding approach than physicians. More research is needed here.

In future research as part of larger studies, HCPs’ approaches in addressing life threat should also need to be directly confronted with patients’ and relatives’ wishes to exchange about this sensitive issue [4]. In fact, patients’ preferences can vary inter-individually, but also intra-individually during the course of the disease [18, 29]. Furthermore, there may be a mismatch between these preferences and the information provided by HCPs, either in terms of excessive, insufficient or inadequate details [18, 30, 31]. Further research will thus help to optimize a patient-centered communication that accounts for the challenges specific to the allo-SCT setting.

Interestingly, all three approaches– reactive, avoiding, and cautious approach– showed correlations with some kind of avoiding attitude towards death. This is in line with other findings, in which death avoidance and fear of death were a barrier to open conversations about dying, among oncologists and oncology nurses [11, 16], and internists and palliative-care physicians [17]. Also, the direct confrontation with death might play a role: In our sample, caring for a higher number of patients in the dying phase was associated with a more proactive, and a less avoiding or reactive approach. All correlations with attitudes towards death were weak; some of them were no longer significant in the HCPs’ subsets, likely due to small sample sizes. This needs further investigation in bigger cohorts, because of the potential impact on HCPs’ ability to adequately address life threat.

Conversations with patients and relatives who overestimate their prognosis was found to be the biggest challenge among our participants, regardless of profession. This finding should inform communication trainings in the allo-SCT setting. This also applies to the wish for mentoring from psycho-oncologists and palliative-care specialists, which emerged as a key insight from our study.

Our survey shows limitations. First, the generalizability of the findings is limited, because of the cross-sectional study design, the small number of participating centers, and because we ignore the exact response rate for the reasons exposed before. Second, the survey consisted mainly of self-developed questions, which allowed us to explore for the first time comprehensively our topic. Self-developed questions present, however, several limitations. Their generalizability is restricted. Their content may over- or under-represent some topics, even though we thoroughly piloted the survey within a multi-disciplinary team to minimize subjectivity. Some formulations might not have been fully comprehensible, or applicable to all participants. For instance, nurses or psycho-oncologists might have felt that some items apply to physicians only, even if we tried to use comprehensive formulations to go beyond the sole question of prognosis disclosure. Third, we did not validate the questionnaire on HCPs’ approaches, because we applied it here for the first time and plan to refine it before validation in larger cohorts. Additionally, the four categories, corresponding to four approaches to address life threat, were built on a theoretical basis and, therefore, can entail subjectivity. However, we mitigated this limitation by conducting an exploratory factor analysis, which statistically confirmed the four approaches. Fourth, the statistical analysis were often conducted on small samples; future studies in larger cohorts are needed to provide more robust and reliable findings.

Nevertheless, our pilot provides important new insights into HCPs’ approaches, challenges and support needs to address life threat. These findings will help to tailor communication trainings in the allo-SCT setting, as part of the Allo-PaS research project [4]. Our study also raises the awareness for potential barriers that may prevent HCPs from communicating adequately about life-threatening conditions. As it strongly highlights differences between professions regarding their approaches, it also contributes to increase understanding within multi-professional SCT teams, and so to promote synergies, when raising existential topics in clinical practice. Overall, our findings will aid in guiding further research on this important topic.

## Electronic supplementary material

Below is the link to the electronic supplementary material.

Supplementary Material 1

## Data Availability

The datasets generated during and analyzed during the current study are available from the corresponding author on reasonable request.
