# Supplementary material for: A cross-sectional survey on health care professionals’ approaches, challenges, and support needs when addressing life threat with recipients of an allogeneic hematopoietic stem cell transplantation and with their relatives
Source: Ann Hematol. 2025 Feb 8;104(1):741–51. doi: 10.1007/s00277-025-06211-6 (PMC11868136; doi:10.1007/s00277-025-06211-6)
Supplement: Supplementary file 1 — Supplementary Material 1 [file 277_2025_6211_MOESM1_ESM.pdf]

Supplementary information

Article title: A cross-sectional survey on health care professionals' approaches, challenges, and support needs when addressing life threat with recipients of an allogeneic hematopoietic stem cell transplantation and with their relatives

Journal: Annals of hematology

Authors: Anne PRALONG, Steffen T. SIMON, Udo HOLTICK, Alinda REIMER, Berenike SCHOERGER, Sukhvir KAUR, Jithmi WELIWITAGE, Martin HELLMICH, Michael HALLEK, Christof SCHEID, Raymond VOLTZ, Marco HERLING

Corresponding author: Dr. Anne PRALONG, University Hospital Cologne, Kerpener Strasse 62, 50924 Cologne, Germany. Email: anne.pralong@uk-koeln.de

Online-resource legends

- Online Resource 1: Self-developed questionnaire
- Online Resource 2: Methods and results of the exploratory factor analysis (EFA)
- Online Resource 3: Correlations between HCPs' approaches to life threat and demographics/personal attitudes towards death as for professional groups
- Online Resource 4: Multiple linear regression analysis with gender and profession as independent variables to predict HCPs' approaches
- Online Resource 5: Significant associations of HCPs' challenges with profession
- Online Resource 6: Significant associations of HCPs' challenges with age and clinical characteristics (*p*-value of Mann-Whitney-U test)
- Online Resource 7: Significant associations of HCPs' support needs with gender and profession
- Online Resource 8: Significant associations of HCPs' needs with age and clinical characteristics (*p*-value of Mann-Whitney-U test)

Online Resource 1: Self-developed questionnaire

1. Addressing the issue "life threat/fear of dying"

How do you address the issue of "life threat/fear of dying" with allo-SCT recipients who have a realistic chance of cure AND a substantial risk of dying, and with their relatives?

Please indicate how much you agree or disagree with the following statements.

		Strongly disagree	Rather disagree	Neither agree nor disagree	Rather agree	Strongly agree	Cannot answer
1.	I struggle to talk to patients/relatives about this topic.	❍	❍	❍	❍	❍	❍
2.	I avoid this topic because it causes me anxiety and/or insecurity	❍	❍	❍	❍	❍	❍
3.	It is not my role to talk about this topic.	❍	❍	❍	❍	❍	❍
4.	I prefer to avoid this topic with patients/relatives, even if I know that they need to talk about it.	❍	❍	❍	❍	❍	❍
5.	I only discuss this topic once the patients/relatives bring it up themselves.	❍	❍	❍	❍	❍	❍
6.	I only address this topic once there is a high risk of death (80-90%, e.g. second allo-SCT).	❍	❍	❍	❍	❍	❍
7.	To give a realistic picture and prevent false hopes, I deliberately talk about this topic in advance of a planned SCT.	❍	❍	❍	❍	❍	❍
8.	During the stressful time of allo-SCT, I avoid this topic in order to give patients confidence.	❍	❍	❍	❍	❍	❍
9.	I repeatedly raise the topic with patients/relatives who unrealistically overestimate the prognosis.	❍	❍	❍	❍	❍	❍
10.	I only address this topic once a planned change of goal of care (curative to palliative) is pending.	❍	❍	❍	❍	❍	❍
11.	I broach the topic with all patients/relatives, unless they expressly do not wish it.	❍	❍	❍	❍	❍	❍
12.	I don't have the time to discuss this topic in depth, so I often don't.	❍	❍	❍	❍	❍	❍
13.	It is important to me to inform all patients well about their risk of dying.	❍	❍	❍	❍	❍	❍
14.	It is important to me to involve my patients' relatives in the conversation about the risk of dying.	❍	❍	❍	❍	❍	❍
15.	I always discuss worst and best-case scenarios with the patients.	❍	❍	❍	❍	❍	❍
16.	After I or others have communicated the risk of death to the patients/relatives, I avoid asking what triggers it in them.	❍	❍	❍	❍	❍	❍

17.	I try to emphasize the chance of cure when talking to particularly worried patients/relatives.	❍	❍	❍	❍	❍	❍
18.	If there is a high risk of death (80-90%, e.g. second allo-SCT), I do not want to weaken the patients'/relatives' hope of cure and therefore deliberately do not talk about this topic.	❍	❍	❍	❍	❍	❍
19.	If there is a high risk of death (80-90%, e.g. second allo-SCT), I try to talk to the patient/relatives to help them prepare for the possibility of dying.	❍	❍	❍	❍	❍	❍

2. Challenges in discussions on the issue "life threat/fear of dying"

When talking to patients with allo-SCT and their relatives, a balance is needed to support the hope of a cure, but at the same time to address the threat to life/fear of dying.

What is challenging for you personally with regard to such conversations? (multiple answers possible)

The own fear or reluctance to talk about dying and death	❍
Talking to patients/relatives who repress the risk of dying	❍
Talking to patients/relatives who overlook the chance of a cure and focus on the risk of dying	❍
Not just to communicate a statistical mortality risk, but to engage in a conversation about threat to life/fear of dying	❍
To be the deliverer of messages of hope and threat to life at the same time	❍
Not to know how much hope and how much information about the risk of dying patients/relatives need	❍
Different views within the team about how much information patients/relatives need about chances of cure and risk of death	❍
To find the right time for conversations about threat to life/fear of dying due to prognosis uncertainty	❍
The own insecurity about initiating and leading conversations about threat to life/fear of dying	❍
None of these/I cannot answer	❍

3. Support needs for discussions on the issue "life threat/fear of dying"

The following is a list of support options that can be helpful when discussing the issue "life threat/fear of dying" with patients with allo-SCT and their relatives.

What support options are, or would be helpful for you personally? (multiple answers possible)

More time for conversations with patients/relatives	❍
Guidance in conversations with (experienced) colleagues	❍
Observation of such conversations by (experienced) colleagues	❍
Training on communication skills	❍
Educational films on communication skills	❍
SOP on communication skills	❍
Pocket guide to communication skills	❍
Joint conversations with psycho-oncologists	❍
Joint conversations with palliative care specialists	❍
None of these/I cannot answer	❍

Online Resource 2: Methods and results of the exploratory factor analysis (EFA)

Methodological steps

- 18 out of 19 items were included into the primary analysis: we omitted the item on time constraints (*"I don't have the time to discuss this topic in depth, so I often don't."*, item 12 in the primary analysis), because this item raises an own theme.
- Extraction was performed by principal axis factoring, based on the correlation matrix and with eigenvalues > 1.0. Varimax was used for rotation. Items were suppressed by a factor loading < 0.4.
- For comparison, we also conducted an exploratory factor analysis by imputing missing values (participants who selected the option "I cannot answer"), but this model did not fit the four approaches defined *a priori* and was rejected.

Results

The following table presents the factor loadings resulting from the final EFA:

	**Factors**			
**Items**	**Proactive approach**	**Avoiding approach**	**Reactive approach**	**Cautious approach**
1. I struggle to talk to patients/relatives about this topic.		**0.680**		
2. I avoid this topic because it causes me anxiety and/or insecurity.		**0.821**		
3. It is not my role to talk about this topic.		**0.498**		
4. I prefer to avoid this topic with patients/relatives, even if I know that they need to talk about it.		**0.673**		
5. I only discuss this topic once the patients/relatives bring it up themselves.	-0.430		**0.530**	
6. I only address this topic once there is a high risk of death (80-90%, e.g. second allo-SCT).			**0.582**	
7. To give a realistic picture and prevent false hopes, I deliberately talk about this topic in advance of a planned SCT.	**0.645**			
8. During the stressful time of allo-SCT, I avoid this topic in order to give patients confidence.			0.419	**0.453**
9. I repeatedly raise the topic with patients/relatives who unrealistically overestimate the prognosis.	**0.722**			
10. I only address this topic once a planned change of goal of care (curative to palliative) is pending.			**0.626**	0.467
11. I broach the topic with all patients/relatives, unless they expressly do not wish it.	**0.532**			
13. It is important to me to inform all patients well about their risk of dying.	**0.703**			
14. It is important to me to involve my patients' relatives in the conversation about the risk of dying.	**0.749**			
15. I always discuss worst and best-case scenarios with the patients.	**0.643**			
17. I try to emphasize the chance of cure when talking to particularly worried patients/relatives.				**0.600**
18. If there is a high risk of death (80-90%, e.g. second allo-SCT), I do not want to weaken the patients'/relatives' hope of cure and therefore deliberately do not talk about this topic (inverse item)	**0.455**			

- N = 75 complete cases
- Bartlett's Test of Sphericity: Chi-square (153) = 607.85, $p < .001$; Kaiser-Meyer-Olkin Measure of Sampling Adequacy: KMO = 0.799

- Four factors resulted from the EFA with eigenvalues > 1.0 and explained 50.8% of the variance.
- The item *"If there is a high risk of death [80-90%, e.g. second allo-SCT], I try to talk to the patient/relatives to help them prepare for the possibility of dying."* (item 19 in the primary analysis) was suppressed after the primary analysis, because of a loading coefficient < 0.4.
- The items *"After I or others have communicated the risk of death to the patients/relatives, I avoid asking what triggers it in them."* (item 16 in the primary analysis) was removed after the primary analysis, because it did not fit theoretically to the factor it was assigned.
- Cross-loadings: The allocation of item 8 to the corresponding factor was conducted based on theoretical considerations.

Online Resource 3: Correlations between HCPs' approaches to life threat and demographics/personal attitudes towards death as for professional groups

	Physicians				Nurses			
	Proactive	Cautious	Reactive	Avoiding	Proactive	Cautious	Reactive	Avoiding
**Demographics**		(*n* = 52)					(*n* = 45)	
Years of clinical practice with allo-SCT patients	n.s.	n.s.	n.s.	n.s.	n.s.	n.s.	n.s.	n.s.
No. allo-SCT patients/year	0.333*	0.288*	n.s.	n.s.	n.s.	n.s.	n.s.	-0.337*
No. patients cared for in the dying phase/year	0.348*	n.s.	n.s.	n.s.	n.s.	n.s.	n.s.	n.s.
- dying phase without outliers (*n* = 95)	0.343*	n.s.	-0.284*	n.s.	n.s.	n.s.	n.s.	n.s.
**Personal death attitudes**								
DAP-R		(*n* = 43)					(*n* = 33)	
Fear of death	n.s.	n.s.	n.s.	n.s.	n.s.	n.s.	n.s.	n.s.
Death avoidance	n.s.	n.s.	0.366*	n.s.	n.s.	n.s.	n.s.	n.s.
Neutral acceptance	n.s.	n.s.	n.s.	n.s.	n.s.	n.s.	n.s.	n.s.
Approach acceptance	n.s.	n.s.	n.s.	n.s.	n.s.	n.s.	n.s.	n.s.
Escape acceptance	n.s.	n.s.	n.s.	n.s.	n.s.	n.s.	n.s.	n.s.
LAP-R		(*n* = 52)					(*n* = 45)	
Death acceptance	n.s.	-0.299*	n.s.	n.s.	n.s.	n.s.	n.s.	n.s.
**Intercorrelations**		(*n* = 52)					(*n* = 45)	
Cautious approach	n.s.				n.s.			
Reactive approach	-0.527***	n.s.			n.s.	0.316*		
Avoiding approach	n.s.	n.s.	n.s.		n.s.	n.s.	0.315*	

Approach subscales built by exploratory factor analysis (Online Resource 2).
The subgroup of psycho-oncologists was not considered, because of small sample size.
* $p \leq 0.05$; ** $p \leq 0.01$; *** $p \leq 0.001$; n.s. = not significant

Online Resource 4: Multiple linear regression analysis with gender and profession as independent variables to predict HCPs' approaches

Variable	B	Standard error	β	t	*p*
Proactive approach					
Males	1.981	1.101	0.160	1.800	0.075
Physicians	6.477	1.113	0.530	5.819	<0.001
Psycho-oncologists	-0.807	1.960	-0.033	-0.412	0.681
Cautious approach					
Males	-0.797	0.438	-0.205	-1.820	0.072
Physicians	0.378	0.442	0.099	0.855	0.394
Psycho-oncologists	-0.542	0.778	-0.071	-0.697	0.487
Reactive approach					
Males	-0.811	0.517	-0.144	-1.568	0.120
Physicians	-3.015	0.523	-0.542	-5.763	<0.001
Psycho-oncologists	-1.863	0.921	-0.168	-2.023	0.046
Avoiding approach					
Males	-0.580	0.578	-0.104	-1.004	0.318
Physicians	-2.094	0.585	-0.380	-3.581	<0.001
Psycho-oncologists	-2.344	1.030	-0.213	-2.276	0.025
Reference group: females, nurses					

Online Resource 5: Significant associations of HCPs' challenges with profession*

Items	Profession (Pearson Chi^2 test or Fisher exact test)			
	**Nurse** (n = 45)	**Physician** (n = 52)	**Psycho-oncologist** (n = 7)	***p*-value**
Not just to communicate a statistical mortality risk, but to engage in a conversation about threat to life/fear of dying	7 (15.6%)$_a$	22 (42.3%)$_b$	$1_{a,b}$	**p = 0.010**
The own insecurity about initiating and leading conversations about threat to life/fear of dying	13_a (28.9%)	5_b (9.6%)	3_a (42.9%)	**p = 0.019**

* Only items with significant differences are listed. They were no significant associations with gender.

a, b: denote a significant group difference if the letters a or b differ between groups. Difference is measured by Bonferroni tests.

Online Resource 6: Significant associations of HCPs' challenges with age and clinical characteristics (*p*-value of Mann-Whitney-U test) *

Items	**Age** (N = 104)	**Years of practice with allo-SCT patients** (N = 104)	**No. allo-SCT patients/year** (N = 104)	**No. dying patients/ year** (N = 104)	**No. dying patients/year without outliers** (n = 95)
To find the right time for conversations about threat to life/fear of dying due to prognosis uncertainty	p = 0.003[a]				
The own insecurity about initiating and leading conversations about threat to life/fear of dying				p = 0.020[b]	

* Only items with significant differences are listed.

[a] Younger age was associated with a more frequent selection of the item.

[b] Lower number of dying patients cared for in the dying phase were associated with a more frequent selection of the item.

Online Resource 7: Significant associations of HCPs' support needs with gender and profession*

Items	Gender (Chi² test)			Profession (Pearson Chi² test or Fisher exact test)			
	**Female** (*n* = 61)	**Male** (*n* = 43)	***p*-value**	**Nurse** (*n* = 45)	**Physician** (*n* = 52)	**Psycho-oncologist** (*n* = 7)	***p*-value**
Training on communication skills	42_a (68.9%)	21_b (48.8%)	**$p = 0.40$**				
Pocket guide to communication skills				6_a (13.3%)	0_b	$0_{a,b}$	**$p = 0.15$**
Joint conversations with psycho-oncologists				39_a (57.4%)	27_b (51.9%)	2_b (28.6%)	**$p < 0.001$**
Joint conversations with palliative care specialists				38_a (84.4%)	28_b (53.8%)	$4_{a,b}$ (57.1%)	**$p = 0.03$**

* Only items with significant differences are listed.

a, b: denote a significant group difference if the letters a or b differ between groups. Difference is measured by Bonferroni tests.

Online Resource 8: Significant associations of HCPs' needs with age and clinical characteristics (*p*-value of Mann-Whitney-U test)*

Items	**Age** (*N* = 104)	**Years of practice with allo-SCT patients** (*N* = 104)	**No. allo-SCT patients/year** (*N* = 104)	**No. dying patients/year** (*N* = 104)	**No. dying patients/year without outliers** (*n* = 95)
Guidance in conversations with (experienced) colleagues	$p = 0.003$[a]	$p < 0.001$[b]		$p = 0.035$[d]	
Observation of such conversations by (experienced) colleagues	$p = 0.025$[a]		$p = 0.009$[c]		

* Only items with significant differences are listed.

[a] Younger age was associated with a more frequent selection of the item.

[b] Less years of practice with allo-SCT patients were associated with a more frequent selection of the item.

[c] Lower number of allo-SCT patients per year was associated with a more frequent selection of the item.

[d] Lower number of dying patients cared for in the dying phase was associated with a more frequent selection of the item.
